# Cerebellar Infarction in a 9 Year Old Child Presenting with Fever and Ataxia: A Case Report

**Published:** 2019

**Authors:** Mohammad VAFAEESHAHI, Nazanin AZIZISHALBAF, Leila TAHERNIA

**Affiliations:** 1 Pediatric Neurology, Pediatric Growth and Development Research center, Institute of Endocrinology and Metabolism, Iran University of Medical Sciences, Tehran, Iran; 2 Pediatric Emergency, Zanjan University of Medical Sciences, Zanjan, Iran; 3 Pediatric Neurology, Tehran University of Medical Sciences, Tehran, Iran

**Keywords:** Cerebellar, Infarction, Ataxia, MRI, Case report

## Abstract

Cerebellar acute ischemic stroke (AIS) can be a complication of minor head trauma, vertebral artery dissection, vasospasm or systemic hypoperfusion. CT scan usually is negative few hours after acute infarction. Magnetic resonance imaging (MRI) is superior to CT scan for posterior fossa lesions and also in acute phase of cerebellar stroke especially in children. Here we report a 9 yr old girl referred to the Pediatric Emergency Room, Moosavi Hospital, Zanjan, Iran in January 2017 presenting with sudden onset of headache and recurrent vomiting, ataxia, and history of 3 consecutive days of fever and malaise. In the report of MRI, there were abnormal low T1 and high T2 signal intensity in left cerebellar hemisphere involving superior and middle cerebellar peduncles. After 4 days of admission, the patient became drowsy, symptoms progressed and transferred to the pediatric intensive care unit (PICU). The patient underwent hemispherectomy surgery of the left cerebellar hemisphere because of acute obstructive hydrocephaly. After 5 months of occupational therapy, the force of her extremities was normal and the ataxia completely disappeared. Childhood acute ischemic stroke although rare can happen with cerebellar involvement. Because in our patient the first brain CT scan was nearly normal and a false negative rate for initial computed tomography (CT) scanning of 60%-80% also contributes to missed and delayed diagnosis of childhood AIS, for every child presenting with acute ataxia without identified cause in addition to CT scan, MRI also being ordered and from the beginning besides other causes, stroke be contemplated as a cause of ataxia.

## Introduction

Childhood Acute stroke (AIS) is a devastating event that can happen with cerebellar involvement occurring in a smaller subset of this group of patients (1). Documented examples of both hemorrhagic and non-hemorrhagic cerebellar stroke in children are available with listed outcomes ranging from occasional death, permanent neurological dysfunction and complete recovery (2). Ataxia, although rare, can be the most prevalent symptom for these children, so early diagnosis is critical. Patients should be separated into two group, the patients with deteriorating condition and the patients with stable or improving condition and the treatment should be selected based on the patient condition (3). Overall scheme of therapeutic approach is to use supportive therapy and anticoagulant therapy. However, the most definite therapy is to reduce ICP with or without surgery according to the patient’s condition (4). 

Here we report a 9 yr old girl presenting with sudden onset of headache and recurrent vomiting, ataxia, and history of 3 consecutive days of fever and malaise.

## Case report

The patient was a 9-yr-old Iranian girl presenting to the Pediatric Emergency Room of Moosavi Hospital, Zanjan, Iran in January 2017, with sudden onset of headache and recurrent vomiting, ataxia and history of 3 consecutive days of fever and malaise. There were left eye ptosis and decrement of left nasolabial fold in patient’s face. The patient had tremor and truncal ataxia. The force of the extremities and deep tendon reflexes were normal in physical examination. Her heart rate was 110 min, blood pressure 90/60 mm Hg and temperature 38.2 °C. The patient’s weight was 21 kg and had 133 cm height and head circumference was 52 cm. The patient had no history of head trauma. 

An informed consent was obtained from her parents in order to publish data as a case report without publishing her name. Ethics Committee of the university approved the study.

**Diagnostic focus and assessment:**

The patient underwent a brain CT scan without contrast in first day of admission preceded by CBC, diff, BUN, Cr, Na, K, Arterial blood gas sampling (ABG) and Urine analysis. On the second day, brain magnetic resonance venography (MRV) and magnetic resonance imaging (MRI) with flair, T1, T2 and DWI sequences and also EEG were ordered, then lumbar puncture with 3 samples was carried out. About 48 h after the MRI the patient became aggressive and had severe headache, nausea and vomiting plus ataxia and delirium, then the patient transferred to PICU and we decided to order the second CT scan and after the results, we asked for urgent neurosurgery consultation and the patient was prepared for brain surgery. The day after surgery the patient underwent brain CT scan without contrast again and laboratory tests were repeated. The patient underwent the last brain CT scan without contrast on thirteenth day and also had an EEG on the same day. We also did a portable echocardiography plus electrocardiography to determine presence of any PFO or ASD or any cardiac problems.

**Figure 1:**
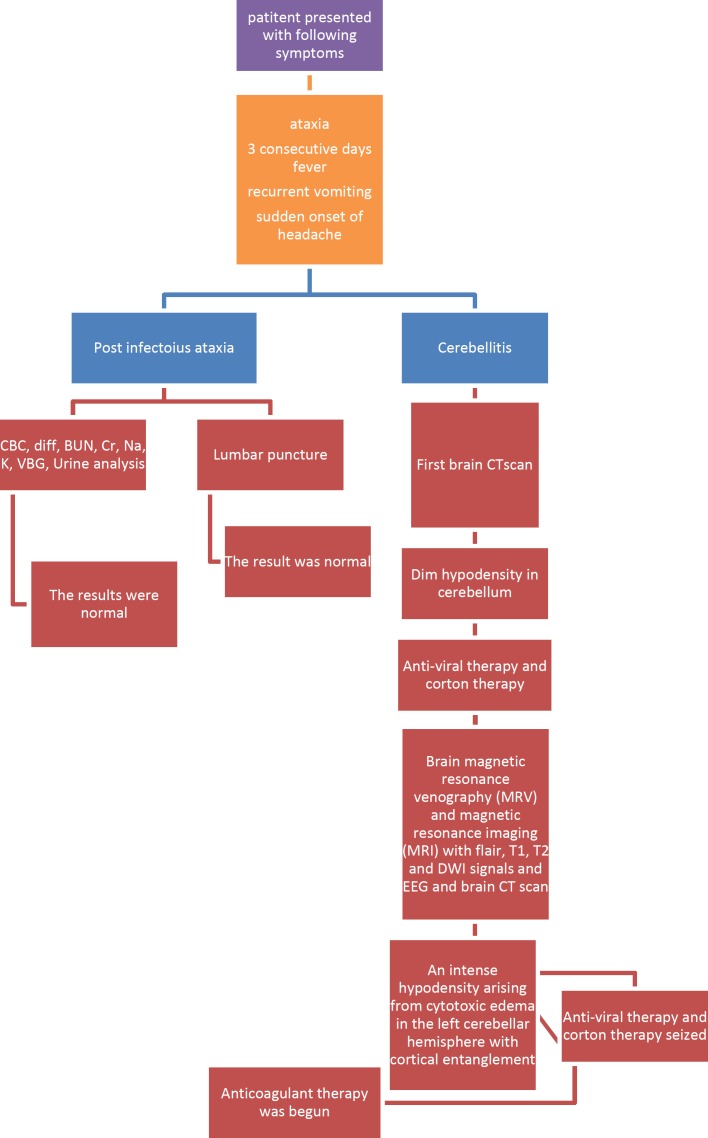
Timeliness of the process of the patient’s follow up.

**Therapeutic focus and assessment: **The patient presented with sudden onset of headache and recurrent vomiting, ataxia, and history of 3 consecutive days of fever and malaise. Our first clinical suspicion was cerebellitis or post infectious ataxia (Figure 1) so we ordered intravenous ceftriaxone, intravenous pantoprazole, and ondansetron for two days. We also ordered acyclovir ampule plus dexamethasone ampule for the patient and after the brain MRI results that were consistent with cerebellar infarction aspirin tablet was prescribed for the patient. On the fourth day (48 h after) because of the patient’s severe headache, intravenous acetaminophen was administered and dexamethasone was held and the patient was transferred to pediatric intensive care unit (PICU) and intravenous phenytoin ampule plus vancomycin ampule were prescribed. After the brain CT, because of obstructive hydrocephaly arising from cytotoxic edema mannitol and dexamethasone ampule were ordered for the patient and the patient became ready for the brain surgery. The next day after the surgery and inserting a drain in the left lateral ventricle, mannitol was discontinued. The day after, phenytoin ampule was held and Diamox tablet (acetazolamide) was ordered. Physiotherapy and occupational therapy of the left extremities begun for the patient and because of constipation lactulose syrup were ordered. 

**Follow-up and outcomes**

**Figure 2:**
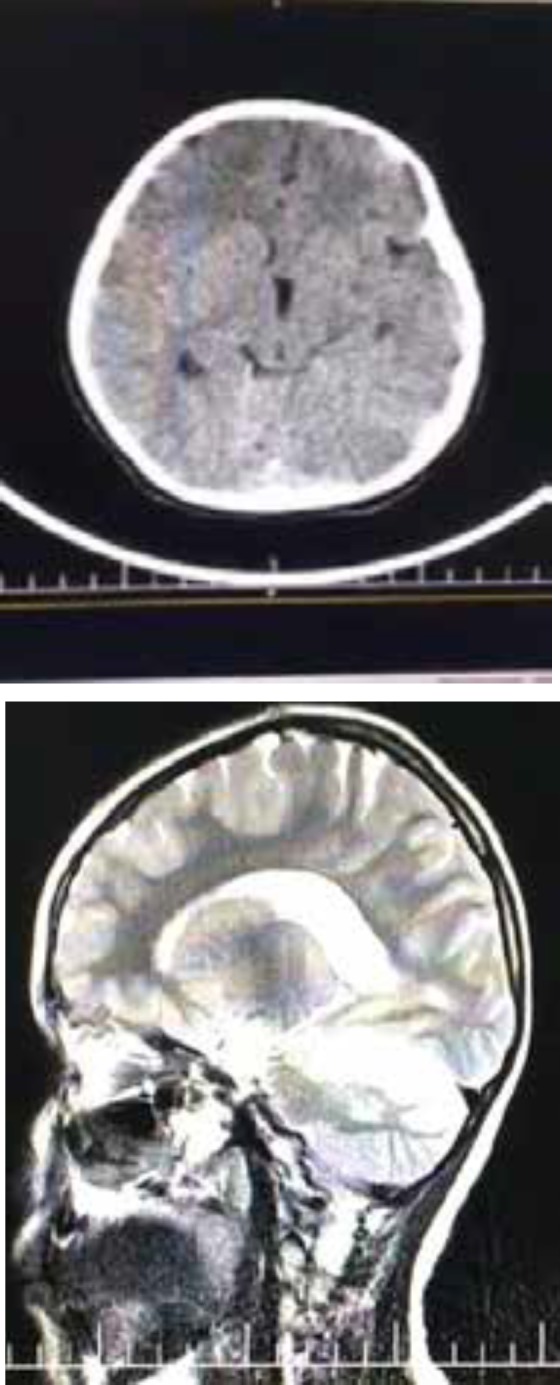
Brain CT scan without contrast (left) on first day showing dim hypodensity in the left cerebellar hemisphere and sagittal plane of brain MRI (right) on second day

The first EEG report was mildly abnormal due to some spike and wave discharges (Figure 2 Left) and in the last EEG report, there were some background attenuations without epileptiform activity (Figure 2 Right). The patient had a lumbar puncture with normal result. The first and the last laboratory data are as follows (Table-1)

**Table 1:** The first and the last laboratory data related to the 9 year-old girl with cerebellar infarction

The first laboratory data	The last laboratory data
BS. Random: normal	BS. Random: normal
BUN: normal	BUN: normal
Cr: normal	Cr: normal
Calcium: normal	Ca: normal
CRP. Quantitative: abnormal (90)	CRP. Quantitative: normal
Na: normal	Na: normal
K: normal	K: normal
ESR.1 hr: normal	ESR.1 hr: normal
WBC: abnormal (13.8)	WBC: normal
HGB: normal	HGB: normal
PLT: normal	PLT: normal
CPK: normal	
PT: normal	
PTT: normal	
INR: normal	
RF: abnormal (27)	RF: abnormal (27)
Lactate: normal	
Ammonia: normal	
AST: normal	
ALT: normal	
C3: normal	
C4: normal	
ANA: normal	
Factor V Leiden: normal	
EBV IgM: normal	
Anti dsDNA: normal	
CH50: normal	
Anti-Phospholipid-IgM: normal	
Anti-Phospholipid-IgG: normal	
Anti-Cardiolipin-IgG: normal	
Anti-Cardiolipin IgM: normal	
Protein C: normal	
Protein S: normal	
Anti-thrombin III: normal	

**Figure 3:**
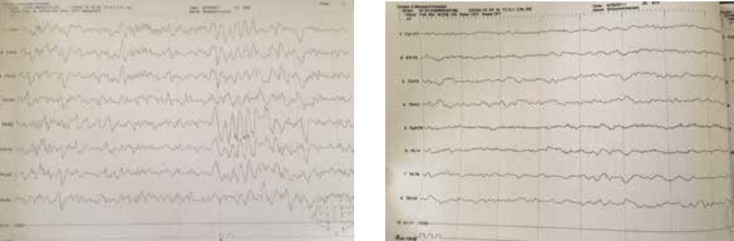
First EEG; Taken while the patient is sleep (left), Second EEG; Taken while the patient is awake (right).

**Figure 4:**
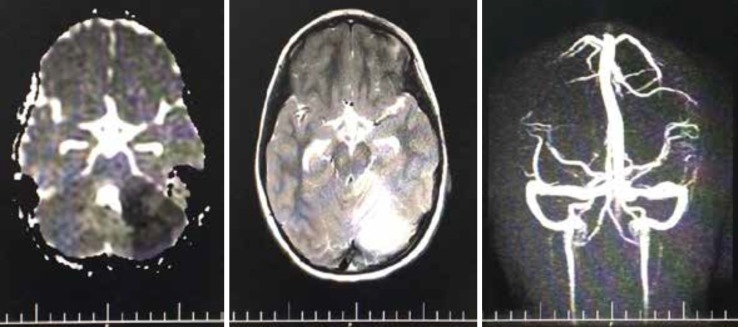
Cerebellar diffusion-weighted image (ADC map) (left) and axial T2 image (middle) showing infarcts at left cerebellar hemisphere on the second day and normal brain MRV (right).

**Figure 5:**
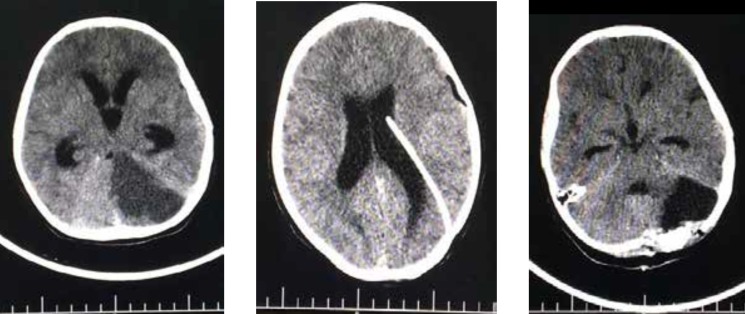
Brain CT scan on fourth day showing infarction on the same area along with obstructive hydrocephaly (left), Brain CT scan after inserting the drain in left lateral ventricle (middle), Brain CT scan after hemispherectomy of the left hemisphere of the cerebellum and extracting the drain (right).

The urine analysis result was normal. The HPLC Amino Acid profile was normal except lower than normal range of arginine (26.8) and higher than normal range of alanine (148.0). We also obtained an Arterial Blood Gas sample from the patient that was normal (Table 1). The echocardiography and electrocardiography results both were normal. Findings on the first brain CT scan were a dim hypodensity in the left cerebellar hemisphere accompanied by a mild generalized ventriculomegaly (Figure 3 Left). The MRV’s result was also normal (Figure 4 Right). In the MRI report, there were abnormal low T1 and high T2 signal intensity in left cerebellar hemisphere involving superior and middle cerebellar peduncles in the same side as well (Figure 3 Right , Figure 4 Middle), restriction was noted in this region. DWI findings were consistent with cerebellar infarction in this region (Figure 4 Left). On the second brain CT scan that was 48 h after the MRI, there was an intense hypodensity arising from cytotoxic edema in the left cerebellar hemisphere with concomitant cortical entanglement exerted pressure on fourth ventricle and had resulted in hydrocephaly, severe ventriculomegaly was also seen in the CT scan (Figure 5 Left) . On the third brain CT which was after the patient’s surgery, there were evidence of craniotomy in occipital bone on the left side and hemispherectomy of the left hemisphere of the cerebellum, the drain in the left lateral ventricle was in its appropriate position (Figure 5 Middle). The last brain CT scan had no new findings and the drain was extracted from the brain (Figure 5 Right). Pathology result of surgical biopsy of brain tissue was cerebellar tissue with small foci of hemorrhage accompanied by presence of vascular malformation, most probably venous angioma.

After 5 months the force of the extremities was normal and the ataxia was completely disappeared. The patient now goes to school and has no problems in daily life activities. The patient is now under treatment with an aspirin 80 mg tablet daily.

## Discussion

Cerebellar acute ischemic stroke (AIS) can be a complication of minor head trauma, vertebral artery dissection, vasospasm or systemic hypoperfusion (2). “Underlying causes of the ischemic infarct cannot be explained in nearly half of cases” (5). Ataxia, although rare, can be the most prevalent symptom for these children, so early diagnosis is critical. CT scan is the most commonly used modality for stroke and is widely available and accurately eliminates acute hemorrhage. In the first hours after acute infarction, CT scan is usually negative (6). MRI is superior to CT scan for posterior fossa lesions and also in acute phase of cerebellar ischemic stroke especially in children. MRI with DWI sequences is more sensitive than conventional MRI. 

Because in our patient the first brain CT scan was nearly normal and a false negative rate for initial computed tomography (CT) scanning of 60%-80% also contributes to missed and delayed diagnosis of childhood AIS, we conclude that for every child presenting with acute ataxia without identified cause in addition to CT scan, MRI also being ordered and from the beginning besides other causes, stroke be contemplated as a cause of ataxia.

**In conclusion, **MRI without any ionizing radiations as the superior modality for showing posterior fossa lesions should be ordered, in addition to CT scan, for every child presenting with ataxia without identified cause, and from the beginning besides other causes, stroke should be contemplated as a cause of ataxia.

## References

[1] Caffarelli M, Kimia AA, Torres AR (2016). Acute Ataxia in Children: A Review of the Differential Diagnosis and Evaluation in the Emergency Department. Pediatr Neurol.

[2] Khair AM, Elseid M, Mohamed K, Al-shami R, Ibrahim K (2014). Cerebellar Stroke in Children, a case report from Qatar & Brief Literature Review. Clin Med Rev Case Rep.

[3] Behzadnia H, Emamhadi M, Yousefzadeh-Chabok S, Alijani B (2015). Posttraumatic Cerebellar Infarction in a 2-year-old Child. Caspian J Neurol Sci.

[4] Wijdicks EF, Sheth KN, Carter BS, Greer DM, Kasner SE, Kimberly WT (2014). Recommendations for the management of cerebral and cerebellar infarction with swelling. Stroke.

[5] Ibrahim Ilker OZ, OZ EB, Şerifoğlu I, Nurullah KA, Erdem O (2016). Cerebellar Infarction in Childhood: Delayed-Onset Complication of Mild Head Trauma. Iran J Child Neurol.

[6] Sığırcı A, Öztürk M, Yakıncı C (2014). Cerebral atrophy and subdural haemorrhage after cerebellar and cerebral infarcts in an 8-month-old child after having been stung by a scorpion. BMJ Case Reports.

